# Pathology and Treatment of Later Stages of Coxitis1Read before the Buffalo Pathological Society.

**Published:** 1891-02

**Authors:** Herman Mynter

**Affiliations:** Professor of Surgery, Niagara University; 195 Franklin Street


Buffalo Medical and Surgical Journal

VOL. XXX. FEBRUARY, 1891. NO. 7.

### Original Communications.

### PATHOLOGY AND TREATMENT OF LATER STAGES OF COXITIS.[1]

BY HERMAN MYNTER, M. D.,
Professor of Surgery, Niagara University.

WHILE our increased knowledge of the pathology of tuberculous joint affections has resulted, in most joints, in clearer indications for operative treatment and improved methods, the same cannot properly be said about tuberculous affections of the hip-joint. We still find the same disagreement between the adherents of conservative and operative treatment, and I scarcely say too much when I state that in the vast majority of cases excision is still made as *ultimum refugium* only. Yet even in these cases a better knowledge of the pathology, and consequently improved operative methods, have been followed by decreased mortality and improved functional results. We are still a long way from performing arthrectomy in the hip-joint as we do in the knee-joint, but the time will surely come when we will cut down upon and remove the local focus, instead of waiting till the whole joint has become disorganized. I propose in this paper first to give a short description of the pathology and some of the symptoms of coxitis, and thereafter particularly discuss the treatment of this the most severe lesion of childhood.

As I said, our knowledge of coxitis has been improved. We formerly believed that tuberculous affections of joints commenced as diffuse inflammations, which went on to destruction of the joints.

Post-mortem examinations were rare except in cases which represented the late stages of coxitis. In these the synovial membrane was always found diffusely diseased, the ligaments and

1. Read before the Buffalo Pathological Society.

the perisynovial tissue changed to a gelatinous, edematous, or fibrous tissue, the joint itself filled with fungous granulations, the cartilages generally ulcerated and shed, leaving the epiphyses in a state of softening and caries. But often we found the cartilages more or less intact, and we therefore believed that the synovitis was the primary lesion, the disease of bones and cartilages secondary. The tuberculous bacillus was unknown, and we supposed a dyscrasia present. Furthermore, all acute infectious diseases were known to be followed occasionally by inflammation of the joints, which always commenced as a synovitis, as in pyemia, puerperal fever, typhus, scarlet fever, etc. It was acknowledged that the inflammation occasionally might commence in the bone, but it was believed that it even then commenced as diffuse inflammation of the medullary tissue in the epiphyses. We overlooked that these diffuse processes, whether in bone or synovialis, were secondary and were the result partly of an infection, partly of reactive and reparative processes.

It is the Germans, particularly the late lamented Professor Volkmann and Professor Koenig, both of whom I quote extensively in this paper, to whom belongs the credit of proving that the fungous or tuberculous joint affections commence, in the majority of cases, as a local focus in the bone, and that the consecutive entrance into the joints of the *materia morbi* from the local focus produces the diffuse inflammations of the synovialis and the epiphyses. That the disease, in a few cases, may commence as a synovitis is not denied. Although post-mortem examinations are rare in the early stages, they are sufficiently frequent to prove this. Nevertheless, the fact remains that the vast majority of cases commence, as Volkmann says, as an osteitis and not as an arthroitis, and more particularly as a circumscribed cheesy or tuberculous osteitis or osteomyelitis. It depends upon circumstances whether the joint later becomes attacked, viz.: whether the products of the inflammation perforate into the joint, or seek the surface. The primary focus, at least in childhood, is always in the bone, either central, or near the periosteum. Generally, only one focus is present, and it is rarely the case that both epiphyses are attacked simultaneously. The focus is generally small, as large as a cherry-pit, or at most as a nut. The neck is the point of predilection in the near neighborhood of the epiphyseal cartilage, but it may commence in the head, the diaphysis, or trochanter major, and perforate the epiphyseal cartilage in order to invade the epiphysis. It may even commence as a chronic osteomyelitis in the cavity of the femur and work its way upward. Primary attacks of the acetabulum, too, are not rare.

The focus presents itself as a little cavity filled with cheesy granulations, bone detritus, and sometimes a sequestrum and surrounded with a pyogenic tuberculous membrane. If the focus perforates into the joint, tuberculous synovitis occurs with very acute symptoms.

If the focus is in the neck or trochanter major, the joint occasionally escapes, the osteitis and abscess being extracapsular, but on the other hand, an acute osteomyelitis, starting in the diaphysis of the femur, may extend into and perforate the joint, producing an acute inflammation but without tuberculous bacilli.

While the osteitic process is going on in the epiphysis and before perforation takes place into the joint, we may discover changes in the joint itself. The synovial membrane, the periosteum, and the periarticular tissue become more or less infiltrated and edematous, slight exudation may occur and a partial obliteration of the joints may take place; little by little the whole synovial membrane may be changed into a granulation tissue, and yet we have no tuberculous arthroitis. When at last the perforation takes place, it is into a half obliterated joint, and the symptoms are, therefore, proportionately less, as a joint reacts the more severely to infectious products when the synovial membrane is physiologically intact, and the less severely the more the synovial membrane has been changed into a granulation-tissue. This partial obliteration we meet particularly in the knee-joint, but less often in the hip-joint. We are, therefore, more apt to meet an acute suppurative arthroitis in the hip, although, occasionally, we may see in the hip a central necrosis of the head and neck being followed by an obliteration of the joint, thereafter destruction of the epiphyseal cartilage and diastasis of the head, which meanwhile has become firmly attached to the acetabulum.

As a rule, the hip-joint is attacked early, as the whole neck and head is inside the synovial capsule, but we may see the joint escape even when the focus starts as a central necrosis in the head or neck. A carious fistule may then be found perforating outward through the trochanter major. This fact is of importance as indicating the way in which such a central necrosis may occasionally be attacked, through trephining of the trochanter and the neck or by ignipuncture.

In the few cases in which the disease commences as a synovitis the final result is the same, destruction of the joint, caries of the bone, etc. The round ligament, which is covered with synovialis, is early attacked and softened, and then disappears.

The osteitic process generally commences at the place of insertion of the round ligament to the head and the acetabulum, and on the neck at the place where the synovialis is attached.

The cartilages become ulcerated by pressure of the granulations (Volkmann's Ulcerative Decubitus), or shed by pressure of the granulation-tissue, meanwhile developed in the dilated Haversian canals. The bones are now in a state of osteoporosis; the head loses its roundness and becomes smaller from pressure, just as the acetabulum enlarges by pressure upwards and backwards or becomes perforated by gradually developed decubitus, and spontaneous dislocation and intrapelvic abscesses result. A periostitis of the ileum occurs as a result of the irritation of the dislocated head, which, under favorable circumstances, may lead to the formation of a new joint. Periarticular abscesses are rarely the result of the breaking down of granulation-tissue in the periarticular tissue. They occur usually from softening and perforation of the capsule itself. They appear often in front, having previously perforated into the iliac bursa. They then extend down on the femur or into the pelvis over the rim. Frequently, too, an abscess perforates above the trochanter minor at the lower margin of the ext. obturator muscle, extending from there to the adductor muscles or backward into the gluteal region, where it then opens near the trochanter major below the gluteus maximus muscle.

A pelvic abscess, from absorption and perforation of the acetabulum, generally follows the iliac muscle downward, but may extend down in the cavum ischio-rectalis, having previously perforated the internal obturator muscle and its covering iliac fascia, and then open into the rectum, perineum or goes down on the femur. It may also perforate backward through incisura ischiadica major.

The symptoms of coxitis are, of course, well known and I shall therefore only call attention to a few minor points. Bonnet has explained the cause of abduction, flexion and rotation outwards in the fact that the capsule can contain more fluid when the limb is in that position. By strongly injecting the capsule in a cadaver, this position is obtained if we previously amputate the femur in the middle. Bonnet thinks that the change from abduction to adduction is simultaneous with perforation of the capsule. I believe that

Koenig's explanation is more correct. He thinks that the position of the limb during the night is the real cause of the deformity, particularly the adduction. He believes that children, who walk around with a coxitis without a crutch or support, by intuition put the limb in abduction and rotation in order to have less strain on the diseased head. They are then obliged to bring the healthy foot forwards quicker in order to save themselves from falling. On the other hand, those patients who present themselves with an early adduction and rotation inwards have either not walked at all or walked with crutches. They have either been in bed for a time, lying on the healthy side, with the diseased limb supported across the healthy one in adduction and rotation inwards, or they have walked with crutches and on purpose raised the pelvis on the diseased side to avoid stepping on the foot. They represent the more severe cases and generally have osteitis of the head of femur. Furthermore, the change from abduction to adduction is almost always contemporaneous with increased intensity of the inflammation, so that the patient is forced to go to bed or use crutches. I myself had a patient (a girl) present herself a couple of weeks ago with the limb in adduction and rotation inwards, who had been sick for only twelve days. The intense pain, the great fixity and the inability to bear any support on the limb so early would necessarily indicate an inflammation on the point of contact, the head, and the adduction and rotation inwards confirmed in my estimation the diagnosis.

Generally speaking, abduction and rotation outwards are most constant in the start; flexion may be wanting. Rotation outwards may occasionally be present alone in the beginning. In the later stadium adduction, rotation inwards and extreme flexion generally occur. Muscular contractions maintain the different positions for a short time only, being followed by further changes such as cicatricial retraction of deep and superficial fascia and fibrous degeneration of the flexior muscles, by which the false position becomes permanent.

In regard to diagnosis little needs to be said. A real shortening without dislocation indicates enlargement of the acetabulum. The swelling in the beginning is most prominent in front, so much so that the ant. cruralis is more prominent. It probably indicates effusion into the joint, and I have several times demonstrated this by puncture of the joint. The swelling of the neck and trochanter indicates osteitic processes in these parts. A considerable uniform elastic swelling behind and above the trochanter major occurs rather late and indicates enlargement of the acetabulum and formation of abscess. The contour of trochanter disappears then so that the tip cannot be felt, and the whole region is strongly prominent on account of the adduction. If the disease occurs primarily with adduction, rotation inwards and severe pain, we may be pretty sure to have an osteitis of the head. In osteitis beginning in the neck and trochanter we will generally find these parts enlarged and tender on pressure. Symptoms indicating destruction of cartilages and the epiphyses, such as starting pains, point to progressive joint affection. Yet it is impossible in a general way to differentiate a primary osteitis from a synovitis. In regard to treatment, I shall only mention, not discuss, such measures as can be found in every surgical text-book, such as rest, extension, derivation, ice, cod liver oil, hygienic measures, etc. Where everything is favorable, as among the rich and educated, results with conservative treatment are generally good. Coxitis may recover in any stage, of course with more or less deformity. In early forms perfect recovery, perhaps with slight contraction and stiffness, may take place. I have myself seen one case, a boy of twelve, after having been in bed for six months with serious deformity, recover so perfectly that for ten years he had absolutely a normal joint. Lately he has had slight symptoms again.

In later stages recovery may take place with true or false anchylosis, or after dislocation, but the usefulness of the limb depends upon the amount of flexion and adduction. Most of our patients, however, come from poor and unsanitary homes, have had poor food, poor air and suffered, in most cases, great neglect. They present themselves in our hospitals emaciated and cachectic, with abscesses and fistulas, and perhaps with symptoms of amyloid degeneration of kidneys and liver, or with Bright's disease. All the resections I have made have been done in such cases, yet the results, both in regard to health and function, are sufficiently encouraging and compare more than favorably with the results of conservative treatment in similar cases. I doubt whether one of them would have been alive today without operation. It must not be forgotten that the statistics of resection must be compared with the statistics of those conservatively treated cases, in which abscesses were present. When we examine the older and newer statistics it must be acknowledged that there is a great decrease of mortality. Leisrink, for instance, gives a mortality of 63 per cent. after resection, of which 22 per cent. succumbed to wound-complications, 21 per cent.

to marasmus, 11 per cent. to phthisis, 2.8 per cent. to diarrhea, 7.5 per cent. to amyloid degeneration and 3.6 per cent. to relapse of caries. A more recent English statistic of 320 cases showed a mortality of 40 per cent. Jacobson has increased Leisrink's statistic of 176 cases to 250 cases and finds a mortality of 40 per cent.

The result of conservative treatment was even worse. Of sixty-three conservatively treated cases in Copenhagen 73 per cent. died and 27 per cent. recovered. An English statistic of 384 conservatively treated cases, in all of which abscesses were present, showed a mortality of 67 per cent., a recovery of 33 per cent.

If abscess was not present, 69 per cent. recovered.

Grosch (1882) found a mortality of 28 per cent. under antiseptic reatment. Koenig states in a recent work, that it is an exception that a patient dies after resection from acute or chronic sepsis.

In spite of the decreased mortality following excision, surgeons still differ in regard to the advisability of conservative or operative treatment. Two English surgeons of large experience, March and Wright, represent well the different opinions. March is strictly conservative and considers excision uncalled for. Continued rest, he says, gives a mortality of only 5 per cent. and 70 per cent. recover with slight lameness and loss of motion. Even when suppuration has occurred he gives a mortality of only 6 or 8 per cent. He considers excision of doubtful benefit even in cases in which the bones have become extensively carious, matter has burrowed widely and the general health has suffered. The operation, he says, will be fatal in 10 per cent. and without improvement in 25 per cent. more. Dr. Wright, on the other hand, with an experience of more than one hundred cases of excision, of which only three died as a result of the operation, strongly advocates excision as soon as external abscesses occur, yes, even before the capsule has been perforated. He maintains that excision cuts short the disease, saves pain, lessens time of treatment and gives a better limb, particularly in hospital cases. Osteomyelitis once established, nothing short of excision can, in his opinion, prevent the progress. Nature can, of course, get rid of the caries and necrosis, but the children who can survive the elimination are few, except among the well-to-do.

The decreased mortality and the better functional results are, as I have said, the results of our increased knowledge of pathology and improved operative methods. Formerly we simply excised the head and perhaps neck and trochanter, but we left the tuberculous synovial membrane and the pyogenic membrane surrounding the abscesses, and we discredited the operation because, as might be expected, suppurations continued or increased and our patients died of marasmus, amyloid degenerations, or tuberculous affections somewhere else. Modern pathology has taught us that coxitis is primarily an osteitis, secondarily a tuberculous synovitis and arthroitis, and that it is necessary not only to remove the bone affections, as we formerly did, but to remove the tuberculous synovial membrane as well. I ascribe my success in excision of the hip principally to the minute and painstaking extirpation of the tuberculous synovial membrane, both of joint and abscesses. If anything is left of that, relapse is sure to occur. Secondarily, I ascribe the success to the antiseptic treatment. If all diseased tissue of bone and synovial membrane is removed we may get healing of the wound by first intention even, just as we see it in operations on knee-joints. I am even inclined to go a step farther than Wright does and advocate still earlier operation in order to remove the local focus and the synovial membrane, if diseased, before diffuse inflammation of the bones has occurred, — in short, make an arthrectomy of the hip-joint instead of an excision. I have so far had no opportunity to carry out this idea, as all my cases have presented themselves in the late stages, or else would not submit to an early operation ; but I find in the *New York Medical Record* of November 1, 1890, the first mention, as far as I know, of a case in which this operation was performed successfully.

Mr. W. H. Battle reports in the London Clinical Society a case of a child, six years of age, in which suppuration of the left hip joint of tuberculous nature was present. The joint was opened by anterior incision (Barker's) and a large quantity of pus removed. The capsule was swollen and softened. A hole was felt in the anterior aspect of the neck, just below (external to) the upper epiphysis.

This was scraped out after enlarging the wound and everting the femur, leaving a mere cylinder of bone to support the head of the femur. The cavity of the bone and the joint were both washed out with chlor. zinc (1–30), and corros. sublimate. The wound was closed with deep catgut sutures without drainage, and within four weeks the child could walk without pain or lameness. The child was subsequently readmitted with a strumuous abscess of the super-condyle region on the right leg, but the left hip-joint appeared in every way perfect. The operation here performed has strictly been an arthrotomy with removal of the local focus, and the operation was probably performed before perforation into the joint had occurred. I do not see any reason why in slightly later cases a typical arthrectomy, *i. e.*, removal of the tuberculous synovial membrane and the local focus, should not be performed, and I hope before long to have the opportunity of trying this method.

I would, of course, first try absolute rest and extension for a few months, open an abscess, if present, or try the iodoform injections, recommended by Mikulicz and Bruns, of Tübingen, on the supposition that prolonged contact of iodoform with tuberculous granulations result in their cure. He uses ten parts iodoform to fifty parts of glycerine and fifty parts of water, and injects it into the abscesses or joint after having first removed the pus by aspiration.

If there is a fungous arthroitis present, he injects 2-4-6 cub. cent. into the granulations. If effusion is present 10-30 grams are injected, after aspiration. In abscesses 50 to 100 grams are injected. Parenchymatous injections are repeated in eight days, joint injections in two to four weeks. Improvement cannot be expected in less than six or eight weeks. The fungous granulations atrophy, the periarticular abscesses shrink into hard nodules. Bruns gives 80 per cent. cures or improvements. If the disease continued in spite of this treatment I should consider myself justified in trying this method. Besides, an early operation would probably prevent tuberculous meningitis. Koenig has pointed out that the dissemination of tuberculous matter during operation is particularly frequent after excision of the hip.

Gerster explains this in the way most excisions are made, the blunt force used, the forcible manipulations which all help to carry the freed caseous matter into the severed veins and lymphatics.

If we were able to diagnosticate with certainty a central necrosis in the head or neck, trephining through trochanter major or ignipuncture though the same would be indicated early, before the joint had become involved, but it is impossible to diagnosticate this condition with certainty, although swelling and tenderness around the neck with absence of distinct joint symptoms may make us suspicious.

There is still one point to consider, *i. e.*, what to do if the coxitis starts in the acetabulum. It is probably impossible to diagnosticate this affection in the start, before intrapelvic abscess has occurred, I have always believed that an intrapelvic abscess, following a coxitis, was a sure sign of affection of the acetabulum, primarily or secondarily, and that it was an absolute indication for resection of the hip as we in no other way could reach the diseased bone and open the abscess. But it seems that there is another way by which we are able from the cavity of the pelvis to expose the inner surface of the acetabulum, open the intrapelvic abscess and eventually resect the acetabulum. Bardenhuer of Cologne has an article in *Deutsche Med. Wochenschrift*, 1890, No. 19, bearing on this point, in which he mentions three cases operated by aid of his symphyseal incision. The operation is performed in the following manner: An incision is made just above the symphysis and along the Poupart's ligaments, extending from the outer third of one Poupart's ligament in a transverse and downward convex direction to the outer third of the other Poupart's ligament, through the skin and subcutaneous tissue. The musculi pyramidales, the anterior fascia of the rectus muscles and these muscles themselves are now cut through, and the insertion of the abdominal muscles to Poupart's ligaments severed. The inguinal canal and the cord are in males drawn outwards.

The fibrous connection between the bladder and the posterior surface of symphysis are now severed as are the ligamenta vesicalia mediana and lateralia, and the bladder sinks backwards. We are now in on the anterior surface of the bladder, the wall of which may be recognized by the yellow-colored longitudinal muscular fibres. The bladder may be attacked through this wound. By keeping next to the posterior surface of the symphysis we may reach the prostate and urethra in men, and laterally the arcus tendineus of the pelvic fascia in both sexes, but it is then necessary to loosen the peritoneum in the iliac fossa commencing anteriorly and working backwards along the linea arcuata interna. We are then on the inner surface of the acetabulum, and may, if necessary, resect it with chisels or only open the abscess.

From theoretical reasons I should think the inner surface of the acetabulum might easier be reached by a semi lunar incision along the tuberositas ischii, opening the excavatio ischio rectalis and working up along the inner surface of the tuberositas ischii. We would only meet one important vessel, art. pudenda communis, which probably could be easily secured in the wound.

Lastly, the question presents itself whether the limb is better after excision or after conservative treatment. Holmes thinks that shortening is generally greater after excision, and the limb less firm and less useful. Motion is more frequently present and more extensive, but the patients often walk more insecurely and with more limp. Jacobson thinks the average results obtained by conservative treatment superior to those following excision, particularly in adults, where we often get flail-joints after excision.

Wright, with his large experience, thinks that excision gives better results and that much shortening depends upon using the limb too early. Some shortening is, of course, always present.

I scarcely consider the question a fair one. It is evident that the best result following excision cannot compare with the best result following conservative treatment, *i. e.*, *restitutio ad integrum*. We must compare those conservatively treated cases, who have got well in spite of abscesses, caries and years of suffering, with those in which, for the same reasons, excision was made. Few get well by conservative treatment, extending during years, without considerable flexion and adduction. To treat such a case demands such continual patience from the side of the parents, the patient and the surgeon, that a good functional result is almost out of the question unless the patient be treated in a hospital, where the surgeon has complete control over the patient and the nurses. The question, to be fair, ought therefore to be as follows: Is a flexed and adducted limb more useful than a shortened limb after excision?

Judging from my own limited experience I believe that excision gives a better functional result and a better looking leg than conservative treatment does in the majority of cases, particularly if you can sever the bone above the trochanter minor. If you are forced to go below the trochanter minor you are very apt to get a flail-joint. I have seen in Buffalo a large number of patients with hip-joint disease, and, as far as I remember, excised the hip eight times. All of these cases except two presented themselves in the third stage of coxitis with extensive carious destruction, large abscesses and broken-down constitutions. Two of the cases I treated under favorable circumstances from the start, but in spite of all precautions they went from bad to worse and excision became necessary. Two of the eight died, one from convulsions during scarlet fever, contracted at the General Hospital immediately after the operation; the other from Bright's disease six weeks after the operation. On the first of these cases I had made an arthrectomy on the knee-joint of the other limb three months previously, and the wound had healed by first intention and the result was excellent. The second case had albuminoid degeneration of liver and kidneys and the operation was done as a last measure. The other six recovered. I can show you four of these cases to-night and will let you judge for yourself whether the results are favorable or not. In order to compare the results with those conservatively treated, I bring before you another patient who was treated in the town under unfavorable circumstances, but in spite of which recovery took place in a flexed and adducted position. His leg, it seems to me, does not compare favorably with the operated ones, so far as appearance is concerned.

195 FRANKLIN STREET.

